# Botulinum Neurotoxin Type A for the Treatment of Benign Prostatic Hyperplasia: Randomized Study Comparing Two Doses

**DOI:** 10.1100/2012/463574

**Published:** 2012-09-10

**Authors:** René Arnouk, Carlos Henrique Suzuki Bellucci, Roberto Benatuil Stull, José de Bessa Junior, Cesar Augusto Malave, Cristiano Mendes Gomes

**Affiliations:** ^1^Department of Urology, Dr. Carlos Arvelo Military Hospital, 1060 Caracas, Miranda, Venezuela; ^2^Uroclínica de Joinville, Rua Otto Boehm, 477—Atiradores, 89201-700 Joinville, SC, Brazil; ^3^Division of Urology, State University of Feira de Santana, 44031-460 Feira de Santana, BA, Brazil; ^4^Division of Urology, School of Medicine, University of Sao Paulo, 01005-010 Sao Paulo, SP, Brazil

## Abstract

*Purpose*. To assess the efficacy and safety of intraprostatic injection of two botulinum neurotoxin type A (BoNT-A) doses for the treatment of benign prostatic hyperplasia (BPH). *Materials and Methods*. Men with symptomatic BPH who failed medical treatment were randomized to receive 100 U or 200 U of BoNT-A into the prostate. The International Prostatic Symptom Score (IPSS), maximum flow rate (Q_max_), post-void residual volume (PVR), PSA levels and prostate volume before injection and after 3 and 6 months were evaluated. Adverse events were compared between the groups. *Results*. Thirty four patients were evaluated, including 17 in the BoNT-A 100 U group and 17 in the BoNT-A 200 U group. Baseline characteristics were similar in both groups. Both doses produced significant improvements in IPSS, Q_max_ and PVR after 3 and 6 months and both doses promoted comparable effects. Prostate volume was affected by 200 U BoNT-A injection only after 6 months of treatment. PSA levels were significantly affected in the 100 U group only after 6 months of treatment. In the 200 U group, PSA levels were significantly decreased after 3 and 6 months. The complication rate was similar in both groups. *Conclusions*. Efficacy and safety of both BoNT-A doses are similar for BPH treatment in the short term followup.

## 1. Introduction

Benign prostatic hyperplasia (BPH) is a nonmalignant enlargement of the prostate that affects men in the adulthood [1]. The purpose of the treatments is to reduce lower urinary tract symptoms (LUTS) and prevent complications such as urinary tract infections, urinary retention, and bladder dysfunction [2, 3].

Several therapeutic options are available, including watchful waiting, pharmacological therapy, and surgical procedures [2–4]. Pharmacological therapy, including 5*α*-reductase inhibitors and alpha-adrenergic antagonists, is the most common treatment for BPH [2, 5–8]. However, improvement of symptoms is often insufficient and the impact on the urinary flow is limited. Moreover, side effects such as dizziness, asthenia, postural hypotension, decreased libido, and erectile dysfunction can limit its use [2, 4]. When pharmacological therapy fails, surgical treatments are usually considered. Despite their high success rates, their invasiveness and potential side effects such as bleeding, retrograde ejaculation, urethral stenosis, urinary incontinence, and erectile dysfunction may be discouraging [4, 6, 9]. Furthermore, 15–25% of the patients who undergo surgery do not have satisfactory long-term outcomes [9] and reoperation is necessary in about 1% annually [6]. 

Consequently, there has been much interest in alternative treatments for BPH and during the last decade, botulinum neurotoxin type A (BoNT-A) has been used to treat LUTS from different etiologies, such as striated sphincter dyssynergia [10, 11], refractory detrusor overactivity [12, 13], and sensory bladder disorders [14]. Recently, the effects of BoNT-A in the prostate have gained attention, and encouraging results in the treatment of BPH have already been published [15–21]. Nevertheless, a number of questions remain unanswered regarding the use of BoNT-A for the treatment of BPH, including the best route of administration, sites of injection, dose, and treatment impact on prostate volume and PSA levels. The objective of this study was to assess the efficacy and safety of two different doses of BoNT-A in the treatment of BPH-associated LUTS.

## 2. Material and Methods

This study was approved by the Local Ethics Committee and all participants gave written informed consent. Over a period of 2 years, men with symptomatic BPH were invited to participate in this prospective study. Inclusion criteria were age above 50 years, persistent moderate to severe LUTS as determined by International Prostatic Symptom Score (IPSS) >8 after medical therapy with at least one alpha-adrenergic antagonist, peak urinary flow rate (Q_max⁡_) of no more than 12 mL/s, and an enlarged prostate gland on digital rectal examination. Exclusion criteria were previous surgery for BPH, urethral stenosis, urinary tract infection, prostate or bladder cancer, pelvic surgery or radiotherapy, neurological diseases, use of any bladder or prostate medications including alpha-blockers, 5-alpha reductase inhibitors or antimuscarinics for the past three months and BPH-associated complications requiring surgical treatment including urinary retention, bladder stone, and bilateral hydronefrosis. 

All patients were bothered by their voiding dysfunction and willing to undergo surgical treatment for it. They underwent further evaluation before treatment, including urinalysis, prostate-specific antigen (PSA), transrectal prostatic and transabdominal urinary tract sonography, free uroflowmetry, and measurement of post-void residual volume (PVR).

Just before the injection procedure, patients were randomized to receive either 100 U or 200 U of BoNT-A. 

### 2.1. Intervention

Injection procedure: with the patient lying on the lithotomy position and under cardiovascular monitoring, local anesthesia was performed with 20 mL of lldocaine 2% gel injected transurethraily and waited for 10-minutes. The injections were performed using a 22Fr rigid cystoscope (Storz, Germany) and a 23-gauge needle (Richard Wolf, Germany). 

The two different BoNT-A (Botox, Allergan, Irvine, CA, USA) doses (100 U or 200 U) were reconstituted with saline 0.9% to a total volume of 5 ml. All patients received five injections of 1 mL of the BoNT-A solution, including two injections in each lateral lobe (one proximal and one distal) and one injection in the medium lobe. The injection depth was 7–10 mm. After the procedure, patients remained under observation until they were able to void spontaneously without hematuria. Oral levofloxacin (500 mg once a day) was administered for five days. 

### 2.2. Followup

Evaluations were performed 3 and 6 months after treatment and included a clinical assessment of LUTS with the IPSS score as well as measurement of peak urinary flow rate, postvoid residual volume, serum PSA levels, and prostate volume. The primary endpoint was improvement of IPSS scores. 

### 2.3. Statistical Analyses

Data were expressed as means ± standard deviation (SD) and range or absolute values and fractions. Intergroup changes from baseline of continuous variables were analyzed with analysis of variance for repeated measurements. Intragroup comparisons were performed using the Student's paired *t*-test. Fisher's test was used for categorical variables. A sample size of 17 in each group has 80% power to detect a difference between means of 3.00 (units in the IPSS score), at a two-tailed significance level of 0.05 or less. Data were processed using commercially available statistical software (GraphPad Prism, version 5.00 for Windows, San Diego, CA, USA).

## 3. Results

We prospectively enrolled 36 patients in this 6-month open-label study. Two patients that failed to return for the follow-up evaluations were excluded. A total of 34 patients completed the study, including 17 in the BoNT-A 100 U group and 17 in the BoNT-A 200 U group. No differences were found at baseline between the two groups, including age, IPSS, Q_max⁡_, PVR, PSA, and PV (Table 1).

**Figure 1:**
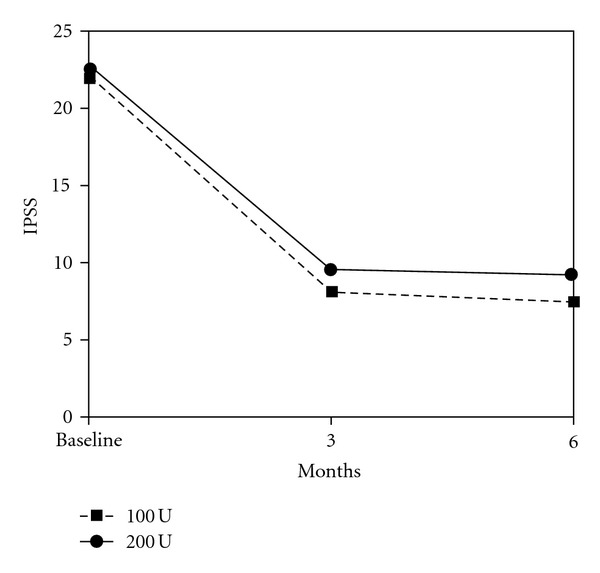
Mean IPSS of 34 patients treated with 100 and 200 U of intraprostatic BoNT-A.

**Figure 2:**
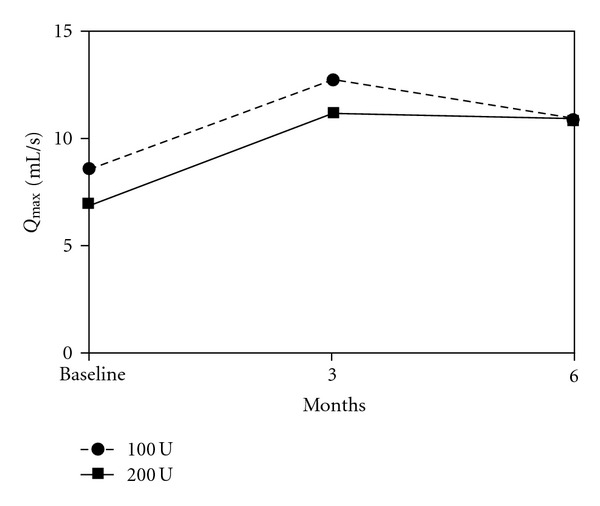
Mean Q_max⁡_ of 34 patients treated with 100 and 200 U of intraprostatic BoNT-A.

**Figure 3:**
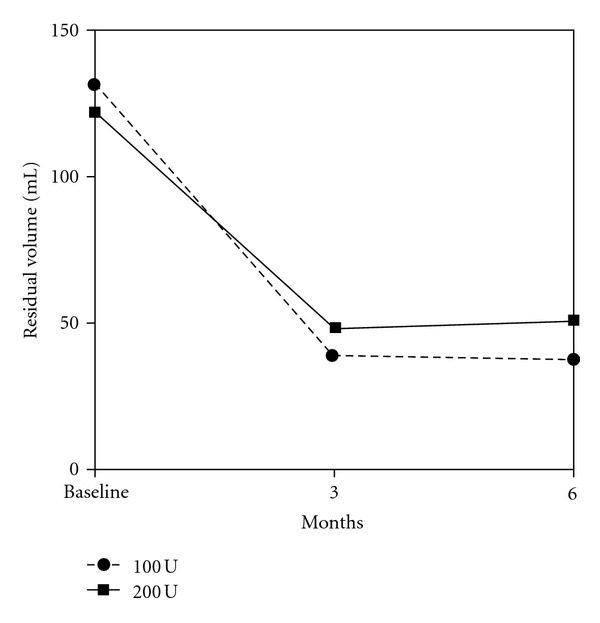
Mean PVR of 34 patients treated with 100 and 200 U of intraprostatic BoNT-A.

The comparison between baseline characteristics and outcome measures 3 and 6 months after treatment are demonstrated in Table 2. Statistically significant changes in IPSS, Q_max⁡_, and PVR were observed at 3rd and 6th months evaluations with both doses of BoNT-A (Figures 1, 2, and 3). 

PSA levels were significantly reduced after six months of BoNT-A 100 U injection but not after three months. In the 200 U group, PSA levels were significantly reduced both after three and six months. 

Prostate volume did not vary significantly in the 100 U group. In the 200 U group, PV was significantly reduced only at the six-month evaluation. 

The impact of intraprostatic injection of 100 U and 200 U of BoNT-A in IPSS, Q_max⁡_, PVR, PSA, and prostate volume was comparable, as seen in Table 2.

Complications and their management are depicted in Table 3. No severe complication was observed. Two (5.8%) patients had transient hematuria requiring bladder irrigation, two (5.8%) had short-term urinary retention, and two had acute prostatitis (5.8%). One patient who initially had mild improvement of symptoms developed urinary retention 5 months after 100 U BoNT-A injection, requiring transurethral resection of the prostate. The complication rate did not differ between the groups (*P* = 0.921). When present, pain was usually mild and no patient needed narcotic analgesics. 

## 4. Discussion

The efficacy of BPH treatment is primarily determined by the magnitude of symptom relief as well as improvement of urinary flow rates. In the present study, both BoNT-A doses promoted significant improvement of symptoms and increased flow rates that continued throughout the followup period of six months.

Maria et al. [15] pioneered BoNT-A injection as a BPH therapy in a double-blind, placebo-controlled trial with 30 men who no longer responded to oral medication and refused surgical treatment. A total of 13 (86.7%) patients in the treated group and 3 (20.0%) in the control group had symptomatic improvement at the 2-month follow-up. Patients in the treatment group had significant improvement in the maximum urinary flow rate, post-void residual urine volume, and IPSS score. Furthermore, PSA levels and prostate volume decreased significantly.

Further studies have documented that intraprostatic BoNT-A injection is an efficient therapy, capable to improve LUTS and Q_max⁡_, as well as to reduce PVR [17, 21]. Our study has also shown these benefits up to 6 months after treatment. Additionally, we recorded that both doses (100 U and 200 U) promoted similar effects. 

One patient treated with 100 U of BoNT-A developed urinary retention five months after the injection. Baseline characteristics of this patient included an IPSS score of 23, maximum flow rate of 3 mL/s, PVR of 135 mL, and a prostate volume 88 mL. These clinical features characterize a severe case of BPH, which might explain why he failed BoNT-A therapy. The patient was treated with transurethral resection of the prostate with a favorable outcome.

The effect of BoNT-A injection on prostate volume is controversial. Experimental studies demonstrated generalized atrophy and apoptosis of glandular and stromal components of the prostate [22–24]. Previous series have shown different rates of prostate volume reduction, ranging from 13 to 54% [15, 16, 19, 21]. Although our study demonstrated benefits in LUTS and flow rates with both doses, a minor (12%) reduction of prostate volume was observed only with the use of 200 U of the neurotoxin. Chuang et al. [17] observed that 12 (29%) of 41 patients treated with of BoNT-A for BPH did not experience reduction of prostate volume, yet seven of these men had significant improvement of IPSS and Q_max⁡_. These data suggest that BoNT-A may act on the dynamic obstructive component of BPH. The neurotoxin was originally thought to act only by inhibiting acetylcholine release at the presynaptic neuromuscular junction [25]. Presently, other mechanisms are known to be involved such as blockage of neuroglandular junctions. It is believed that it also promotes a decrease of norepinephrine release from sympathetic endings, leading to the consequent reduction of alpha-1A adrenoceptor stimulation [26]. Furthermore, in an experimental model using rats, a dose-dependent decrease in the expression of alfa-1A adrenoceptors was demonstrated [27]. This is another possible mechanism affected by BoNT-A treatment, which may promote a decrease in density of alfa-1a adrenoceptors, which are known to be increased several-fold in BPH [28]. 

Different doses (range from 100 to 300 U) have been studied in several series, but there is a lack of consistency in some studies [15–21]. Some authors suggest that prostate size might influence the dosage [17, 21], but whether larger prostates require higher doses has actually not been tested. Moreover, there is no evidence whether the severity of LUTS influences the optimal dose. To the best of our knowledge, it is the first study comparing two different doses of BoNT-A. 

The procedure has been performed by transperineal, transrectal and transurethral approaches. We chose the transurethral route because the vast majority of urologists are trained on cystoscopic procedures and also because it permits direct vision and injection in the transition zone of the prostate. Kuo [21] firstly described the cystoscopic approach using light general anesthesia or sedation. We have demonstrated that it is feasible to perform this technique with local anesthesia. Pain, when present, was mild and no patient required narcotics in the postoperative period. 

Complications were uncommon in our series and seemed to be related to the urethral manipulation rather than a direct result of BoNT-A injection. Gross hematuria, although conservatively managed, was observed in 2 (5.8%) patients. Transient urinary retention was observed in 2 (5.8%) patients, and treated conservatively with an indwelling catheter for 5 days. Two patients (5.8%) developed acute prostatitis. As mentioned, these adverse effects seem to be associated with the route of administration of BoNT-A, since they are potential complications of any procedure requiring urethral instrumentation. Series that adopted the transperineal or transrectal approach did not report such complications [22–27]. 

Given the vascular nature of the prostate, systemic absorption of the toxin could occur. The doses used in intraprostatic injection of BoNT-A are well below the presumed fatal dose. To our knowledge, no systemic complications have been reported after intraprostatic injections, even with 300 U of BoNT-A [18].

Kuo injected 200 U of BoNT-A in the prostate of ten poor surgical candidates, with BPH and urinary retention or large postvoid residual volume. All patients improved spontaneous voiding after treatment. Both voiding pressure and postvoid residual volume were significantly decreased after treatment. Total prostate volume was significantly reduced and maximal flow rate was significantly increased after treatment [21]. Our study showed that the cystoscopic route can be performed using local anesthesia, which may contribute to further decrease treatment risks. Despite our encouraging results, large-scale, randomized studies with long-term followup are needed to determine the best delivery route, sites of injection, suitable dosing as well as the long-term effects. Studies comparing its cost effectiveness with that of pharmacological and traditional surgical modalities are also necessary. Finally, due to its action on cell proliferation, apoptosis, and afferent pathways, the potential role of BoNT-A in the treatment of prostate cancer and chronic pelvic pain may be studied in the future. 

## 5. Conclusion

Transurethral intraprostatic BoNT-A injection is a simple and safe therapy for men with symptomatic BPH. Efficacy and safety of 100 U and 200 U BoNT-A doses are similar.

## Figures and Tables

**Table 1:** Baseline characteristics of patients treated with intraprostatic BoNT-A.

	BoNT-A 100 UI	BoNT-A 200 UI	*P* value
*N*	17	17	
Age (yr)	66 ± 8.8	67 ± 10.0	0.59
IPSS	22.0 ± 6.4	22.0 ± 6.9	0.72
Q_max_ (mL/s)	8.6 ± 3.1	8.4 ± 3.1	0.87
PVR (mL)	131.8 ± 65.0	121.1 ± 73.7	0.88
Prostate volume (mL)	42.3 ± 18.5	43.1 ± 19.7	0.83
PSA (ng/dL)	3.9 ± 4.1	4.1 ± 2.7	0.86

IPSS: international prostatic symptom score, Q_max_: maximum urinary flow rate, PVR: postvoid residual volume.

**Table 2:** Mean and percentage change from baseline of International Prostatic Symptom Score (IPSS), maximum urinary flow rate (Q_max⁡_), pos-void residual volume (PVR), PSA levels and prostate volume after 3 and 6 months of treatment.

	BoNT-A 100 U	Change %	BoNT-A 200 U	Change %	*P* value^†^
IPSS					
Baseline	22.1 ± 6.4		22.8 ± 6.9		
3rd month	8.0 ± 4.4*	*−64%*	9.5 ± 4.2*	*−58%*	0.767
6th month	7.5 ± 4.3*	*−66%*	9.2 ± 3.4*	*−60%*	0.657
Q_max⁡_ (mL/s)					
Baseline	8.6 ± 3.1		8.4 ± 3.2		
3rd month	12.8 ± 3.6*	*49%*	11.2 ± 4.8*	*33%*	0.564
6th month	10.9 ± 3.4*	*27%*	11.4 ± 3.2*	*36%*	0.927
PVR (mL)					
Baseline	131.8 ± 65.0		121.1 ± 73.7		
3rd mo	39.1 ± 33.5*	*−70%*	48.1 ± 24.4*	*−60%*	0.466
6th mo	38.5 ± 31.2*	*−69%*	51.7 ± 24.7*	*−57%*	0.311
PSA (ng/dL)					
Baseline	3.9 ± 4.1		4.1 ± 2.7		
3rd mo	3.2 ± 3.3 ns	*−18%*	3.0 ± 2.1*	*−27%*	0.426
6th mo	3.0 ± 2.5*	*−23%*	2.7 ± 1.7*	*−34%*	0.421
Prostate volume (mL)					
Baseline	42.3 ± 18.5		43.1 ± 19.7		
3rd mo	38.9 ± 16.1 ns	*−8%*	39.8 ± 17.7 ns	*−8%*	0.961
6th mo	38.6 ± 16.6 ns	*−9%*	37.8 ± 15.5*	*−13%*	0.561

^†^Statistical significance between groups (intergroup comparison) (Student's *t*-test).

Statistical significance within groups: *P < 0.05 versus baseline, ns (not significance versus baseline).

(Repeated measures ANOVA and Dunnett's posttest).

**Table 3:** Complications after intraprostatic botulinum toxin injection for BPH.

Complication	*n* (%)	Post-op	Treatment	Outcome
Hematuria	2 (5.8%)	Immediate	Bladder irrigation for 2 hours	Resolution
Transient urinary retention	2 (5.8%)	Immediate	Foley catheter for 5 days	Resolution
Persistent urinary retention	1 (2.9%)	5 months	TURP	Resolution
Prostatitis	2 (5.8%)	Immediate	Amikacin	Resolution

TURP: Transurethral resection of the prostate.
